# Efficacy and safety of Elevate^®^ system on apical and anterior compartment prolapse repair with personal technique modification

**DOI:** 10.1590/S1677-5538.IBJU.2016.0233

**Published:** 2017

**Authors:** Daniele Castellani, Vikiela Galica, Pietro Saldutto, Giuseppe Paradiso Galatioto, Carlo Vicentini

**Affiliations:** 1 Department of Life, Health & Environmental Sciences, University of L'Aquila, Urology Unit, “Giuseppe Mazzini” Hospital, Teramo, Italy

**Keywords:** Pelvic Organ Prolapse, Surgical Mesh, Vagina, Surgical Procedures, Operative

## Abstract

**Aim:**

To evaluate the effectiveness and safety of Anterior Elevate^®^ mesh kit system (AES) in woman with symptomatic stage 3 or 4 anterior and/or apical pelvic organ prolapse (POP).

**Materials and Methods:**

This retrospective, monocentric, single surgeon study enrolled between May 2010 and January 2013 fifty-six woman experiencing symptomatic anterior vaginal prolapse with or without apical descent (POP-Q stage 3 or 4). All women received a AES and 7 (12.5%) received a concomitant transvaginal hysterectomy. Primary endpoint was anatomic correction of prolapse; success was defined as POP-Q stage ≤ 1 or asymptomatic stage 2. Secondary endpoints were quality-of-life (QOL) results and patients' safety outcomes, which were assessed by 3 validated self-reporting questionnaires at baseline and annually: ICIQ-UI short form, ICIQ-VS and P-QOL. All patients completed 2-years and 28 women 3-years of follow-up. Surgical approach was modified in women with uterus, moving the two-propylene strips anteriorly around the cervix itself crossing one another, so the left will take place in the right side and the right on the opposite. This modification was made in order to better support the uterus.

**Results:**

Vaginal mesh exposure was present in 3 (5,3%) patients. Very good anatomical outcomes were seen, with one (1,8%) failure at 6-months, 4 (7,1%) at 1-year, 6 at 2-years (10,7%). Statistically significant improvements were seen in the ICIQ-VS and P-QOL questionnaires throughout follow-up.

**Conclusion:**

Our data suggest that AES is a minimally-invasive transvaginal procedure to repair anterior and apical POP, with good evidence related to mid-term safety and efficacy.

## INTRODUCTION

Pelvic organ prolapse (POP) occurs when there is a disruption of the natural supporting structures of the pelvic organs, often with impaired function of the pelvic floor musculature. The loss of these normal attachments and the dynamic support of the pelvic floor result in the descent of one or more pelvic structures including the bladder, the rectum, the uterus and cervix, or the vaginal cuff and the small bowel in case of previous hysterectomy. Even if there is a lack of epidemiological studies of the natural history, incidence and prevalence of POP, it is widely accepted that 50% of women will develop prolapse, but only 10 to 20% of those seek evaluation for their condition (1). POP has become a major health concern, as it may affect 50% of women over age 50 (2) and the lifetime risk of needing surgery for prolapse or urinary incontinence by 80 years of age has been reported as high as 19% (3). Many women with clinically evident POP on physical examination may be asymptomatic. When symptomatic, they complain of bothersome symptoms that can be divided in vaginal urinary, defecatory and sexual. Treatment for POP is based upon symptom bother, patient expectation, and quality of life impact. The direct costs of POP in the United States has been estimated at U.S. $1billion, with similar costs expected throughout the developed countries (4). Given the increasing time and resources that will be required for POP surgery in the future, it is paramount to perform effective, durable, cost-effective interventions with minimal morbidity. The failure of traditional repairs has led to the use of graft materials, particularly synthetic mesh, to augment prolapse repair in an attempt to improve success and durability. In the last decade, several mesh kits have been developed and commercialized to repair POP through a vaginal approach (5). All these procedures have gained popularity, because of their minimally-invasive approach and low morbidity rate. Despite that, the Food and Drug Administration issued a Public Health Notification in October 2008 to inform physicians and patients of adverse events related to vaginal reconstructive surgical use of synthetic mesh and to provide recommendations on how to mitigate risks and counsel patients appropriately (6). A wide spectrum of potential complications exists with the use of transvaginal mesh in POP surgery, even severe complications, such as fistula formation, mesh erosion into adjacent organs, and death. In 2010, after a 15-years of experience on transvaginal POP and stress urinary incontinence (SUI) mesh surgery, we started using the Elevate^®^ Anterior and Apical prolapse system (AES) kit (American Medical Systems, Minnetonka, MN) to repair anterior and apical compartment pro-lapse. In women with uterus we decide to make a change to the standard technique, moving the two-propylene strips anteriorly around the cervix itself crossing one another. This approach could give a better support to the uterus left in place. The aim of the study presented in this paper was to evaluate the effectiveness and safety of AES in women with symptomatic stage 3 or 4 anterior and/or apical POP.

## MATERIALS AND METHODS

This is a retrospective medium-term study on safety and efficacy of AES in apical and anterior compartment prolapse correction. The study had been reviewed and approved by a certified Ethical Board. Inclusion criteria were symptomatic primary or recurrent anterior and/or apical compartment prolapse stage 3 or greater, according to pelvic organ prolapse quantitative (POP-Q) system (7). Exclusion criteria were known hypersensitivity to synthetic materials, pelvic cancer or chemo-therapy 1-year before enrollment, previous pelvic irradiation, previous mesh surgery, restricted leg motion, uncontrolled diabetes, immune suppression or use of immune modulators. All patients signed an informed consent form and the change in the licensed mesh technique have been entailed a specific consent form. All women were evaluated with medical history, clinical examination, cough test, 24 hour-pad test, smear test, urodynamics and multiple self-reported validated questionnaires. POP was staged by senior surgeon (C.V.) using the POP-Q system (7) and occult SUI was evaluated using a pessary placement (8) during urodynamics evaluation. Urodynamics were performed in accordance with International Continence Society (ICS) recommendation (8). Subjective and quality—of-life (QOL) outcomes were assessed by three validated self-reporting questionnaires at baseline and annually. Urinary incontinence was evaluated using the International Consultation on Incontinence questionnaire on urinary incontinence (ICIQ-UI) short form (9), vaginal and sexual symptoms using International Consultation on Incontinence questionnaire on vaginal symptoms (ICIQ-VS) (10) and prolapse-quality of life questionnaire (P-QOL) (11). All data were routinely collected for all patients. The primary outcome of this study was anatomic correction of prolapse; success was defined as POP-Q less than or equal to stage 1 or asymptomatic stage 2. Secondary outcomes were QOL results and patient safety outcomes. Complications were reported according to Clavien and Dindo Classification of surgical complications (12) and to The International Urogynecological Association (IUGA)/ICS joint terminology and classification of the complications related directly to the insertion of prostheses and grafts in female pelvic floor surgery (13). Between May 2010 and January 2013 fifty-seven women met the criteria and were enrolled in the study, held at Urology Unit, University of L'Aquila, Teramo Hospital, Italy. One patient was lost at follow-up. Fifty-six patients were available for analysis. Slings were not performed at the same time in any patient with SUI, because we prefer a staged mesh surgery. Mid-urethral slings were offered subsequently only in patients who needed. Statistical analysis was performed using the Student t-test. Qualitative date are shown as mean±SD. Statistically significantly difference is considered as p<0.05.

### Surgical Technique

AES kit contains a shaped mesh with two—self anchoring tips and two-propylene strips with a self-fixating tip at their top. Distally the mesh has to be fixed in the obturator foramen using the attached self-fixating tips. Proximally two strips have to be attached to the sacrospinous ligament using the needle present in the kit, which are assembled with the mesh. All patients received antibiotics prophylaxis with ceftriaxone 1g intravenously 30 minutes prior surgery. All procedures were performed by single experienced pelvic surgeon (C.V.), in a dorsal lithotomic position and under spinal anesthesia. Surgery is different in woman with uterus respect of those with post—hysterectomy vault prolapse. In latter group the AES is placed in the standard fashion, according to the instructions for use. Conversely in patients with uterus in place, surgery begins with an incision at level of posterior vaginal wall, followed by a sharp and blunt dissection bilaterally towards the sacrospinous ligament, where the combined elements are placed in the standard fashion. After that, a second incision and dissection are performed in the anterior vaginal wall. After a blunt dissection of the cervix, the two-propylene strips are moved anteriorly around the cervix itself crossing one another, so the left will take place in the right side and the right on the opposite. This personal technique modification can be watched in a video clip available online in the journal website. At this point, surgery is equal in both groups; the distal part of the mesh is anchored using the needle, which drives the self-fixating tips to the obturator internal muscle. The mesh is distally fixed with two tension-free vicryl 2/0 sutures at the level of the bladder neck and proximally to the uterosacral ligaments or their residual part in patients without uterus. Finally, both propylene strips are inserted to the open eyelets and adjusted in a tension-free manner using the locking eyelets. Cystoscopy is performed to rule out any bladder injury. Surgery ends with closure of vaginal wall incisions with a double vicryl suture, 2/0 internally and 0/0 externally, positioning a 16-Fr indwelling catheter and vaginal packing. In case of stage 2 posterior compartment prolapse a simple colpoperineoplasty is performed.

## RESULTS

Fifty-seven women underwent transvaginal anterior POP repair using a polypropylene mesh AES between May 2010 and January 2013. One patient has been lost at follow-up. Statistical analysis was performed in 56. Patient's characteristics and demographics at baseline are shown in Table-1. Mean operative time was 47.3 (±8) minutes. Twelve patients had previous surgery for anterior prolapse and 33 had a previous hysterectomy. Seven women underwent a concomitant hysterectomy for large uterine volume due to fibromatosis. No bladder injury was seen. Urethral catheter and vaginal packing were removed in the first post-operative day. Only one patient went to retention, most probably because she had a voluminous bladder diverticula; she was managed with self-intermittent catheterization and regained spontaneous micturition on day 5. Ultrasound post-voiding urine residual (PVUR) was performed in each patient twice daily; significant PVUR (≥100mL) was not present in the remaining patients. All patients were discharged on post-operative day 2. No major bleeding was observed: mean drop in post-operative hemoglobin was 1.7±0.6g/dL. Post-operative complications, according to the Clavien-Dindo classification (12), in the first month were grade I (intravenous analgesics and anti-emetics) in ten cases, and grade III-a (vaginal infected hematoma with wound dehiscence, requiring drainage in local anesthesia) in one. This complication can be classified as 3CbT2S1 according to IUGA/ICS terminology and classification of the complications related directly to the insertion of prostheses and grafts in female pelvic floor surgery (13). Transient buttock pain was reported in 4 (7.1%) of the patients in the first week post-operatively and it disappeared spontaneously. All patients were examined 6-weeks after surgery, 6 months and then annually with urine culture and pelvic examination according to POP-Q. ICIQ-UI—short form, ICIQ-VS and P-QOL questionnaires were self-administered annually. Post-operative results are shown in Table-2. All patients completed a 2-years and 28 3-years of follow-up. Anatomical results were excellent because failure (defined as symptomatic POP stage 2 or stage ≥3) was seen in 1 (1.8%) woman at 6 months, in 4 (7.1%) at 1-year and in 6 (10.7%) at 2-years. At 3-years of follow-up only 3 patients out of 28 (10.7%) had POP ≥3 stage (data not showed in Table-2). Vaginal mesh exposure, defined as grade III-b (12) or 3BT3S1 (13) complication, was seen in 3 patients (5.3%) during the first year of follow-up. Questionnaires outcomes showed statistical significant improvement of symptoms and QOL domain except for incontinence (Table-3).

**Table 1:** Baseline demographics and characteristics.

		Number	%
Age, mean ± SD		69±5.3	
BMI, mean ± SD		26.5±4.5	
Vaginal deliveries, Mean ± SD		2.6±1.4	
Menopausal status		56	100
Prior hysterectomy		33	58.9
Prior prolapse surgery		12	21.4
SUI before surgery		21	37.5
Urge incontinence		7	12.5
Sexually active		40	71.4
	Dyspareunia	13	23.2
Sexually inactive		16	28.6
Stage 3 anterior prolapse		22	39.2
Stage 4 anterior prolapse		19	33.9
**Apical prolapse**			
	Vault	23	41
	Uterine	10	17.8
	Enterocele	5	8.9
Concomitant hysterectomy		7	12.5

**Table 2:** Anatomical POP-Q results and complications at follow-up.

		6 weeks	6 months	1 year	2 years
		n (%)	n (%)	n (%)	n (%)
Stage 1 POP		3 (5.3)	6 (10.7)	9 (16)	12 (21.4)
Stage 2 POP		1 (1.8)	5 (8.9)	5 (8.9)	6 (10.7)
	symptomatic	0 (0)	1 (1.8)	1 (1.8)	2 (3.5)
Stage 3 POP		0 (0)	0 (0)	2 (3.5)	3 (5.3)
Stage 4 POP		0 (0)	0 (0)	1 (1.8)	1 (1.8)
*De novo* SUI		5 (3)	4 (7.1)	5 (8.9)	5 (8.9)
Persistent SUI		10 (17.8)	10 (17.8)	10 (17.8)	11 (19.6)
*De novo* urgency		2 (3.5)	3 (5.3)	8 (14.3)	6 (10.7)
Urge incontinence		0 (0)	1 (1.8)	8 (14.3)	6 (10.7)
Persistent dyspareunia		2 (3.5)	5 (8.9)	4 (7.1)	5 (8.9)
*De novo* dyspareunia		3 (5.3)	3 (5.3)	5 (8.9)	5 (8.9)
Vaginal mesh exposure		0 (0)	1 (1.8)	2 (3.5)	0 (0)
Positive Urine Culture		8 (14,3)	10 (17,8)	7 (12,5)	9 (16)

**Table 3:** Subjective ICIQ-UI short form, ICIQ-VS and P-QOL outcomes at baseline and after AES implant.

		Preop	1 year	2 years	3 years
		Mean ± SD (range)	Mean ± SD (range)	Mean ± SD (range)	Mean ± SD (range)
**ICIQ-UI-short form** (max=21)		10.2±2.1 (0–16)	6.3±1.8 (2–12) p>0.11	6.1±1.5 (3–11) p>0.1	6.5±1.8 (3–12) p>0.18
**ICIQ-VS (max)**					
	Vaginal symptoms (53)	18.5±8.6 (9–37)	3.2±3.1 (0–20) p<0.011	4.2±5.9 (0–28) p<0.13	4.4±5.9 (0–21) p<0.14
	Sexual matters (58)	18.9±16.5 (0–42)	2.5±7.4 (0–25) p<0.01	6±12.5 (0–26) p<0.02	5±11.8 (0–36) p<0.02
	QOL (10)	4.7±3.9 (0–10)	0.8±1.4 (0–6) p<0.01	0.5±1.5 (0–7) p<0.01	0.7±1.5 (0–7) p<0.01
**P-QOL (max)**					
	General health	53±9.5 (50–75)	26 ± 3.2 (12–44)p<0.023	24±2.5 (11–35) p<0.022	23±3.4 (12–37) p<0.021
perception(100)					
	Prolapse impact (100)	93±3.2 (67–100)	5±2.3 (0–12) p<0.001	10±3.2 (0–17) p<0.004	12±2.5 (0–18) p<0.004
	Role limitations (100)	61±7.4 (51–85)	2±2.3 (0–7) p<0.001	3±3.5 (1–5) p<0.001	2±3.2 (1–5) p<0.001
	Physical limitations (100)	62±7.8 (33–83)	2±1.8 (0–17) p<0.001	3±2.4 (0–19) p<0.001	3±3.1 (0–20) p<0.001
	Social limitations (100)	58±6.5 (22–56)	2±0.8 (1–11) p<0.001	3±1.2 (1–12) p<0.001	3±1.7 (1–12) p<0.001
	Personal relationship (100)	67±12.1 (38–100)	1±0.5 (1–3) p<0.001	2±0.8 (1–4) p<0.001	2±0.7 (1–4) p<0.001
	Emotions (100)	55±5.6 (45–89)	1±0.8 (1–3) p<0.001	2±1.2 (1–4) p<0.001	2±1.6 (1–4) p<0.001
	Sleep/energy (100)	25± 5.7(17–41)	2±0.8 (1–5) p<0.001	3±0.9 (1–5) p<0.001	3±1 (1–5) p<0.001
	Severity measures (100)	42±7.5 (34–65)	4± 4.2 (1–14) p<0.002	6±3.8 (2–15) p<0.003	6±4.1 (2–16) p<0.003

**Preop =** preoperative. Not statistically significant vs preoperative (p>0.05) Statistically significant vs preoperative.

## DISCUSSION

Traditional anterior colporrhaphy for repair of anterior prolapse has an estimate risk of recurrence between 30-50% (14, 15). Randomized controlled trials and recent meta-analysis showed superior anatomical outcomes in mesh repair compared to anterior colporrhaphy (14, 16, 17). Nowadays more than 40 implants are available on the market (18) even with little evidence on their safety and efficacy related to mid- and long—term. Indeed, the FDA warned in 2011 regarding serious complications associated with transvaginal placement of surgical mesh and reinforced the basis that surgeons should perform prolapse repair only if they are adequately subspecialized in this area (19). The AES is a relative new kit composed of a type I polypropylene mesh with bilateral anterior and posterior graft arms for anchoring them to the obturator foramen and sacro-spinous ligament, respectively. This kit has two major advantages; first its fixation is easy to perform via self-fixating tips, avoiding blind trocar passage through the obturator and perirectal fossa seen with alternative mesh kit techniques. Secondly it seems to fit perfectly anterior and apical prolapse surgical repair, because it is well known that most anterior-compartment prolapse is associated with apical prolapse (20). Crossing of the strips of the mesh anteriorly to the cervix is different from the standard technique. We made this change because we believe that it could better support uterus. Our data show results of the AES for the repair of anterior and apical vaginal wall prolapse with a minimum of 2-years of follow-up in 56 patients and 3-years in 28. It is possible, hence, to find several important findings; first of all, the AES appears to be a safe and minimally invasive procedure with very low incidence of associated adverse events. There was no bladder and rectal injuries. Only 3 vaginal mesh exposure were identified and were treated surgically with excision of the exposed vaginal area. Transient buttock pain was reported in 4 (7.1%) of the patients in the first week post-operatively and it disappeared spontaneously. The mechanism of this transient pain is likely due to local entrapment of pudendal branches such as the perforating cutaneous nerve (21). Secondly, our mean operative times is shorter (47.3 min) than reports of abdominal (221–225 min) and robotic (226–328 min) sacrocolpopexy (22, 23). Third, our series demonstrate very good anatomical outcome, with one (1.8%) failure at 6-months, 4 (7.1%) at 1-year, 6 at 2-years (10.7%). At 3-years follow-up only 3 patients out of 28 (10.7%) were POP ≥3 stage. Our 1-year anatomic results were similar to other transvaginal mesh procedure; Vaiyapuri (24) reported in his series of Prolift^®^ a cure rate of 92.1%, Jacquetin (25) 81.6% of success rate in TVM technique. The anatomic result remained stable for the next two years (89.2% at 2-year of follow-up and 87.4% at 3-year of follow-up). Our results are consistent with other AES series recently published (26–27). Both Rapp (26) and Huang (27) have 90% of anatomical success rate at 2-years follow-up. Anatomic failure is present in our series at one-year follow-up and it remains almost the same during the next two years; no patients required a second surgery so far. Last but not least, the current series demonstrates excellent subjective outcomes; ICIQ-VS and P-QOL questionnaires demonstrated statistically significant improvements not only in vaginal and sexual symptoms, but also in QOL at each follow-up visit. Regarding preoperative SUI we prefer, as previously mentioned, a staged surgery in these patients, because restoring pelvic organ support has cured SUI in 10 women out of 21 who had preoperative SUI as showed in Table-2 (11 patients with persistent SUI after 2-years of follow—up). We performed a secondary sling procedure only in patients asking for it (8 women) after at least one year of follow-up. Our results showed that AES is a minimally-invasive transvaginal procedure to repair anterior and apical POP, with good evidence related to mid-term safety and efficacy. Further studies are indeed needed to confirm the long-term results.

## Abbreviations

POP: = Pelvic organ prolapse
AES: = Elevate^®^ Anterior and Apical prolapse system
SUI: = stress urinary incontinence
POP-Q: = pelvic organ prolapse quantitative
ICS: = International Continence Society
QOL: = quality-of-life
ICIQ-UI: = International Consultation on Incontinence questionnaire on urinary incontinence
ICIQ-VS: = International Consultation on Incontinence questionnaire on vaginal symptoms
P-QOL: = prolapse-quality of life questionnaire
